# Air pollution exposure and cardiometabolic risk

**DOI:** 10.1016/S2213-8587(23)00361-3

**Published:** 2024-02-01

**Authors:** Sanjay Rajagopalan, Robert D Brook, Pedro R V O Salerno, Brendan Bourges-Sevenier, Philip Landrigan, Mark J Nieuwenhuijsen, Thomas Munzel, Salil V Deo, Sadeer Al-Kindi

**Affiliations:** University Hospitals, Case Western Reserve University School of Medicine, Cleveland, OH, USA (Prof S Rajagopalan MD, P R V O Salerno MD, B Bourges-Sevenier BS); Division of Cardiovascular Diseases, Department of Internal Medicine, Wayne State University, Detroit, MI, USA (Prof R D Brook MD); Program for Global Public Health and the Common Good, Boston College, Boston, MA, USA (Prof P Landrigan MD); Centre Scientifique de Monaco, Monaco (Prof P Landrigan); ISGlobal, Centre for Research in Environmental Epidemiology, Barcelona, Spain (Prof M J Nieuwenhuijsen PhD); Department of Cardiology, University Medical Center of the Johannes Gutenberg University, Mainz, Germany (Prof T Munzel MD); German Center of Cardiovascular Research, Partner-Site Rhine-Main, Germany (Prof T Munzel); Louis Stokes Cleveland VA Medical Center, Case Western Reserve School of Medicine, Cleveland, OH, USA (S V Deo MD); DeBakey Heart and Vascular Center, Houston Methodist, Houston, TX, USA (S Al-Kindi MD)

## Abstract

The Global Burden of Disease assessment estimates that 20% of global type 2 diabetes cases are related to chronic exposure to particulate matter (PM) with a diameter of 2·5 μm or less (PM_2·5_). With 99% of the global population residing in areas where air pollution levels are above current WHO air quality guidelines, and increasing concern in regard to the common drivers of air pollution and climate change, there is a compelling need to understand the connection between air pollution and cardiometabolic disease, and pathways to address this preventable risk factor. This Review provides an up to date summary of the epidemiological evidence and mechanistic underpinnings linking air pollution with cardiometabolic risk. We also outline approaches to improve awareness, and discuss personal-level, community, governmental, and policy interventions to help mitigate the growing global public health risk of air pollution exposure.

## Introduction

A changing environment and factors such as sedentary habits, seismic shifts in agrarian practices, and widespread availability of nutrient-dense foods have irrevocably altered the balance between energy intake and expenditure.^1^ The International Diabetes Federation reports that 10·5% of adults worldwide, or 537 million individuals, have type 2 diabetes, with almost half of these individuals unaware that they have the condition.^2^ By 2045, projections show that 783 million individuals will be living with type 2 diabetes, with 75% of these cases occurring in low-income and middle-income countries.^2^ There is strong accruing evidence linking chronic exposure to pollutants in air, soil, and water with cardiometabolic health.^3,4^ Air pollution is by far the most dominant environmental risk factor for health in general, and is responsible for over 9 million annual deaths globally, most of which are due to cardiovascular causes.^3,5,6^ A recent Global Burden of Disease study estimated that a fifth of worldwide type 2 diabetes is attributable to chronic fine particulate matter (PM_2·5_) exposure.^7^ People with type 2 diabetes are likely to be more susceptible to adverse health effects from PM_2·5_, which further amplifies pollution-related cardiovascular risk.^3,7^ This Review aims to provide an up to date summary of the epidemiological and mechanistic evidence linking air pollution with diabetes, outline approaches to improve awareness, and review much-needed personal-level and policy interventions to help mitigate this enormous and growing global public health risk. Unless otherwise stated in this Review, PM_2·5_ exposure in animals almost always means inhalational exposure to concentrated ambient PM_2·5_. Recently, preliminary evidence linking air pollution and type 1 diabetes has also emerged.^8^ However, given the strength of evidence for the association between type 2 diabetes and air pollution, as well as its dominant global prevalence and relevance to cardiovascular disease, the focus in this Review is type 2 diabetes.

## Environmental underpinnings of type 2 diabetes

Type 2 diabetes is not a single disease, but rather a group of conditions broadly categorised by a single diagnostic criterion—hyperglycaemia. Subtypes of type 2 diabetes that are phenotypically and genetically distinct have been noted, with differential susceptibility to target organ complications such as cardiovascular disease, nephropathy, neuropathy, and retinopathy.^9^ Phenotypic manifestations of type 2 diabetes can include individuals with excess visceral adiposity, lean individuals, and individuals with predominant liver and skeletal muscle deposition of fat. This phenotypic diversity complicates genetic attribution of type 2 diabetes, as each feature might have differential genetic susceptibility, and also complicates the interpretation of environmental studies, including those on air pollution. The preponderance of studies nevertheless suggests a major contribution of the environment in shaping susceptibility to type 2 diabetes, defined by glycaemic criteria, and its complications.^10^ External factors in the built and natural environment, lifestyle and behaviours, social environment, chemical exposures, and internal factors (such as the microbiome, stress, and sleep duration), which collectively constitute the exposome, have also been shown to affect susceptibility to type 2 diabetes.^11–15^

## Air pollution source components and relationship to metabolic disease

Air pollution continues to be the world’s most pronounced environmental health threat. Worldwide, poor air quality accounts for 93 billion days lived with illness and more than 6 million deaths each year, although some estimates put the number of deaths well above 9 million.^3^ The highest burdens of air pollution mortality are often in large, urban cities.^16^ The total economic cost of air pollution exceeds US$8 trillion, or roughly 6·1% of global annual gross domestic product.^17^ The major sources of modern-day air pollution in high-income countries include fossil fuel combustion. However, globally, other sources (eg, desert dust and volcanic ash) play a major role in air pollution, with growing concern related to wildfire-associated air pollution, which is markedly increasing in the USA and globally due to the effects of climate change.^5^ Air pollution chemistry and sources have been reviewed extensively in previous reports, but most health effects are associated with particulate matter fraction, with the particles being categorised by size fraction into ultrafine particles (<0·1 μm [PM_0·1_]), fine particles (diameter ≥0·1 μm to 2·5 μm [PM_2·5_]), and coarse particles (diameter >2·5 μm to 10 μm [PM_10_]).^18^ Coarse particles originate from crustal materials and agricultural and industrial practices, and can dominate air particulate composition in certain environments. PM_2·5_ exposure occurs primarily from fossil fuel combustion from power generation and transportation, although exposures from wildfire smoke and other climate events are increasingly common and can dominate many environments for months.^19–21^

Air pollution is a complex mixture of gasses, particles, and liquids that are continually changing and interacting with each other and natural atmospheric gasses. Although PM_2·5_ mass has rightfully attracted attention as a target for regulation and study, more than 98% of the mass of air pollution is from gases or vapour-phase compounds, such as CO, non-methane hydrocarbons or volatile organic carbons, NO_2_, NO, O_3_, and SO_2_.^3,5,22^ In addition to primary pollutants formed directly by combustion, many other pollutants are produced primarily through chemical reactions in the atmosphere and are known as secondary pollutants. Examples include PM-associated sulphate, nitrate, and ammonium, and many of the organic compounds within PM_2·5_, including various inorganic and organic acids and volatile organic compounds. However, the current understanding of each individual component’s effect on human health is insufficient. Fresh combustion PM, such as from traffic-related pollutants, is largely composed of ultrafine particles. Although there is some evidence linking ultrafine particles with cardiometabolic diseases, definitive data are difficult to acquire due to the short-lived nature of ultrafine particles, their high spatial variability, the focal locations of exposures (such as near roadways), and their high correlation to other environmental exposures (eg, noise).

### Global PM2.5 concentrations and burden of disease

**Figure 1:**
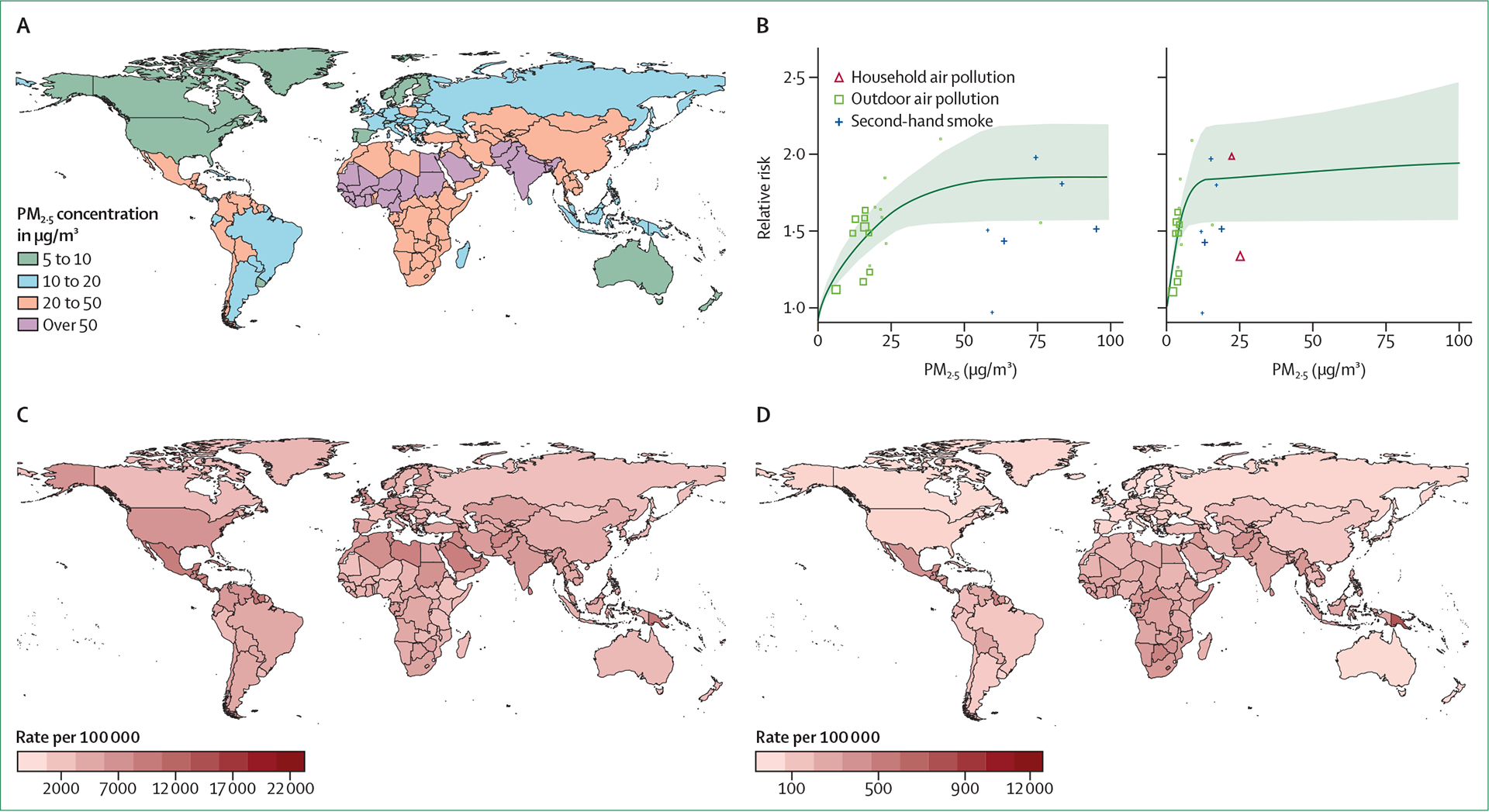
Global PM_2·5_ and diabetes data for 2019 From Global Burden of Disease data.^7^ (A) Ambient PM_2·5_ concentrations. (B) Exposure–response curve of PM_2.5_ and diabetes, shown across PM_2·5_ 0–100 μg/m^3^ and 0–500 μg/m^3^. (C) Age-standardised prevalence of type 2 diabetes. (D) Type 2 diabetes age-standardised disability-adjusted life years associated with particulate matter pollution. PM_2·5_=particulate matter smaller than 2·5 μm.

Over 99% of the global population live in areas with PM_2·5_ exposure exceeding WHO air quality guidelines (<5 μg/m^3^ annually) (figure 1A).^23^ Pollution-attributed health effects disproportionately affect heavily populated countries, especially China and India.^3,4^ In 2019, only 0·18% of the global land area and 0·001% of the global population had an annual exposure to PM_2·5_ that was lower than the WHO limit of 5 μg/m^3^.^23^ The mean global annual population-weighted PM_2·5_ concentration for 2000–19 was estimated at 32·8 μg/m^3^.^23^ Areas with higher than average Black, Asian, and Hispanic or Latino populations are routinely exposed to higher than average PM_2·5_ concentrations, with evidence of significant health effects even at lower PM_2·5_ concentrations.^24,25^

### Dose response of air pollution and incident type 2 diabetes

A study evaluating PM_2·5_ exposure and the development of diabetes in a group of 1 729 108 US veterans with no history of diabetes was one of the first to use an integrated exposure–response function to estimate risk.^26^ An increase of 10 μg/m^3^ in long-term PM_2·5_ exposure was associated with an increased risk of diabetes (hazard ratio [HR] 1·15, 95% CI 1·08–1·22) after adjusting for 57 variables comprising individual-level health, sociodemographic factors, physical environment, and health behaviours. At levels of exposure typical for the USA, ambient PM_2·5_ was calculated to contribute to about 3·2 million incident cases of diabetes and 206 105 deaths.^26^ In an updated analysis, using data from ambient air pollution, household air pollution, and second-hand smoking, the Global Burden of Disease investigators extended the range of their analysis to all known global concentrations of PM_2·5_ (figure 1B).^7^ The shape of the exposure–response curve was curvilinear, with a more robust association at lower PM_2·5_ levels and an attenuation of the relationship above 50 μg/m^3^ (HR approximately 1·5–1·7). Applying the exposure–response curve to the global population in 2019, the study estimated that 20% of all diabetes-related deaths and disability-adjusted life years (DALYs) globally were attributable to PM_2·5_ (13·5% to ambient air pollution and 6·5% to household air pollution).^27^ The shape of the exposure–response curve has helped provide a credible explanation for why even low doses of second-hand smoke or air pollution are deleterious from a cardiometabolic perspective. The global type 2 diabetes age-standardised prevalence is illustrated in figure 1C, and figure 1D depicts the PM_2·5_-associated type 2 diabetes DALY rates in 2019, obtained from the Global Burden of Disease study.^28^

## Epidemiological evidence linking PM2·5 and cardiometabolic disease

### Insulin resistance

Several studies (appendix p 2–4) have shown that short-term exposure (days) to air pollution is associated with insulin resistance, a primary predisposing condition to type 2 diabetes across various concentrations and age groups. The development timeline in humans is rapid, occurring within hours to days. This association has been noted in children and adults across a broad range of ages for PM_2·5_ and traffic-related pollutants (such as NO_2_). However, it is important to note that air pollutants often correlate spatiotemporally; thus, it might be challenging to isolate the effects of one pollutant in epidemiological studies.

### Air pollution and diabetes

Several meta-analyses (appendix p 5–9) have reviewed the effect of air pollution exposure on diabetes incidence and prevalence; in general, air pollution exposure was associated with an average increase in incident diabetes (type 2 diabetes) that generally ranged between 10–25%. The association between air pollution and diabetes is higher in men, groups with low socioeconomic status, and individuals with other comorbidities.^29^ A meta-analysis of 31 eligible cohort studies demonstrated an increased risk for gestational diabetes associated with increased PM_2·5_, PM_10_, SO_2_, and NO_2_ concentrations^.30^ The presence of obesity might also modify the risk for type 2 diabetes.^31^ Diabetes is a complex disease that takes years to develop, with an additional period of delayed diagnosis. Given these facts, epidemiological findings that have categorised type 2 diabetes at the time of clinical recognition are underestimates, with the real burden of disease probably substantially higher.

### Air pollution and obesity

In a meta-analysis to estimate the effects of childhood exposure to air pollutants on weight, 15 studies were included.^32^ Both obesity and BMI were significantly associated with annual PM_10_, PM_2·5_, and PM_0·1_ exposure. The estimate was strongest for PM_2·5_, with an odds ratio (OR) of 1·28 for weight (95% CI 1·13–1·45) and 0·11 for BMI (0·05–0·17) per 10 μg/m^3^ increment in exposure.^32^ In a cohort consisting of male US veterans, correlations were significant between PM_2·5_ exposure and obesity (HR 1·08, 95% CI 1·06–1·11) and weight (1·07, 1·06–1·08).^33^ Several nationwide cohort studies in adults demonstrated a link between air pollution exposure and obesity and other metabolic syndrome components at higher levels of air pollutants.^34,35^ Longitudinal studies have been largely negative at lower annual PM_2·5_ concentrations (median ≤20 μg/m^3^).^36–38^ Given that air pollution concentrations have been decreasing over time owing to regulation, even when PM_2·5_ exposure was used as a time-dependent variable to adjust for decreasing PM_2·5_ concentrations, levels higher than 25 μg/m^3^ were associated with higher risks of obesity and components of metabolic syndrome in at least two large national cohorts.^38,39^

### Air pollution and diabetes-related mortality

Several studies have explored the association between PM_2·5_ and diabetes-related mortality at high and low air pollution levels. In a study of 2·1 million adults living in Canada, at low PM_2·5_ exposure levels (8·7 μg/m^3^; SD 3·9), there was an increase in the risk of diabetes-related mortality per 10 μg/m^3^ of PM_2·5_ (HR 1·49, 95% CI 1·37–1·62), which persisted well below levels of 5 μg/m^3^.^40^ A study of 52 061 participants in the Danish Diet, Cancer and Health cohort found an association between increased risk for diabetes-related mortality and long-term exposure to NO_2_ (mortality rate ratio 1·31, 95% CI 0·98–1·76 for 10 μg/m^3^ increase after adjustment for confounders).^41^ Positive associations between diabetes-related mortality and exposure to short-term NO_2_, CO, and SO_2_ concentrations have been reported in a time series study conducted in Montreal.^42^ In the American Cancer Society, Cancer Prevention Study II cohort, a 10-μg/m^3^ increase in PM_2·5_ was associated with an 18% increase in diabetes-related mortality.^43^

### Susceptibility and vulnerability

The health effects of air pollution from a cardiometabolic perspective are pronounced among low-income and minoritised racial and ethnic groups populations, partly due to increased exposures.^24,25^ Evidence also shows that the benefit of lowering PM_2·5_ levels, even to well below the current US National Ambient Air Quality Standards standard of 12 mg/m^3^, might also be larger in these populations.^44,45^ Although some studies have shown that people with diabetes might have greater adverse health effects compared with those without diabetes at similar PM_2·5_ exposure levels, this is not a consistent finding because people with diabetes show substantial variation in health (eg, duration of disease and risk factor profile) and medication treatment.^45^

### Cardiovascular risk

The evidence that links acute and chronic PM_2·5_ exposure with fatal and non-fatal cardiovascular events, including stroke and heart failure, has been reviewed extensively.^3,5^ A small number of studies have investigated PM_2·5_ and cardiovascular risk in type 2 diabetes. In a subset of the 2001 Canadian Census Health and Environment Cohort, association between PM_2·5_ and cardiovascular mortality was stronger in people with diabetes (HR 1·51, 95% CI 1·39–1·65, per 10 μg/m^3^) compared with those without (1·20, 1·16–1·25), suggesting increased PM_2·5_ susceptibility in patients with diabetes.^46^ In a study from China including 15 477 individuals with multipollutant ascertainment (eg, PM_1·0_, PM_2·5_, PM_10_, SO_2_, NO_2_, and O_3_), diabetes status potentiated the relationship between pollutants and cardiovascular disease in all exposures.^47^

### Renal and ophthalmic risk

Emerging evidence suggests that PM_2·5_ is associated with chronic kidney disease in populations both with and without diabetes. Bowe and colleagues described a linear relationship between long-term PM_2·5_ exposure, incident chronic kidney disease, and progression to end-stage renal disease that was independent of the presence of diabetes.^48^ In another cohort study of more than 2·4 million US veterans with median 8·5 years of follow-up, Bowe and colleagues found that a 10 μg/m^3^ increase in PM_2·5_ was associated with increased odds of diabetes and chronic kidney disease.^49^ Diabetes mediated 4·7% (4·3–5·7) of the association of PM_2·5_ with incident estimated glomerular filtration rate (eGFR) less than 60 mL/min per 1·73 m^2^, 4·8% (95% CI 4·2–5·8) with incident chronic kidney disease, 5·8% (5·0–7·0) with 30% or greater decline in eGFR, and 17·0% (13·1–20·4) with end-stage renal disease or 50% or greater decline in eGFR.^49^ A longitudinal study of people with diabetes in Taiwan associated urinary albumin-to-creatinine ratio measurements with PM_2·5_ and CO exposure.^50^ In an analysis of 469 933 deaths due to chronic kidney disease across 2304 counties in the USA, the association between PM_2·5_ and chronic kidney disease mortality was strongest among counties with the highest social deprivation index (β0·70, 95% CI 0·49–0·92I), versus counties with the lowest social deprivation index (β0·49, 0·41–0·56).^51^

Few studies have examined the association between PM_2·5_ and diabetic retinopathy risk. In a study of newly diagnosed diabetes in Taiwan (2003–12), which included 579 patients, every 10 μg/m^3^ increase in PM_2·5_ was associated with a 29% increase in the odds of retinopathy (OR 1·29, 95% CI 1·11–1·50), whereas every 10 μg/m^3^ increase in PM_10_ was associated with a 37% increased risk in developing retinopathy (1·37, 1·17–1·61).^52^

### Non-alcoholic fatty liver disease

In a nationwide cross-sectional analysis in the USA in patients with non-alcoholic fatty liver disease (n=269 705) there was a positive association between ambient PM_2·5_ exposure and the odds of non-alcoholic fatty liver disease (adjusted OR 1·24 per 10 μg/m^3^ increase, 95% CI 1·15–1·33) among hospitalised patients.^53^ Despite the PM_2·5_ exposure levels being much higher than those in the USA and Europe, similar results were also demonstrated using nationwide data from China.^54^

### Air pollution exposure during pregnancy and diabetes

A recent meta-analysis investigated the association between exposure to PM_2·5_ and gestational diabetes at various stages of pregnancy.^55^ For the full pregnancy, the pooled relative risk (RR) was 1·074 (95% CI 1·001–1·152). Moreover, during the first and second trimesters, RRs of 1·015 (1·000–1·031) and 1∙021 (1·006–1·035), respectively, were observed. However, no significant association was found between preconception exposure to PM_2·5_ and gestational diabetes (1·013, 0·990–1·038). In another study from Taiwan that explored the potential effect-modifying capacity of PM_2·5_ exposure on development of type 2 diabetes in women with and without gestational diabetes, the odds ratio of diabetes per interquartile range increase in PM_2·5_ was 1·31 (1·22–1·41), with the risks of developing type 2 diabetes with exposure to increased PM_2·5_ levels being significantly higher in the gestational diabetes group than the non-gestational diabetes group.^56^ Despite this evidence, there are still substantial knowledge gaps that require further investigations to explore the ramifications of PM_2·5_ exposure during different gestational periods and the potential long-term cardiometabolic effects.

## Towards mechanistic understanding of air pollution-mediated metabolic risk

**Figure 2:**
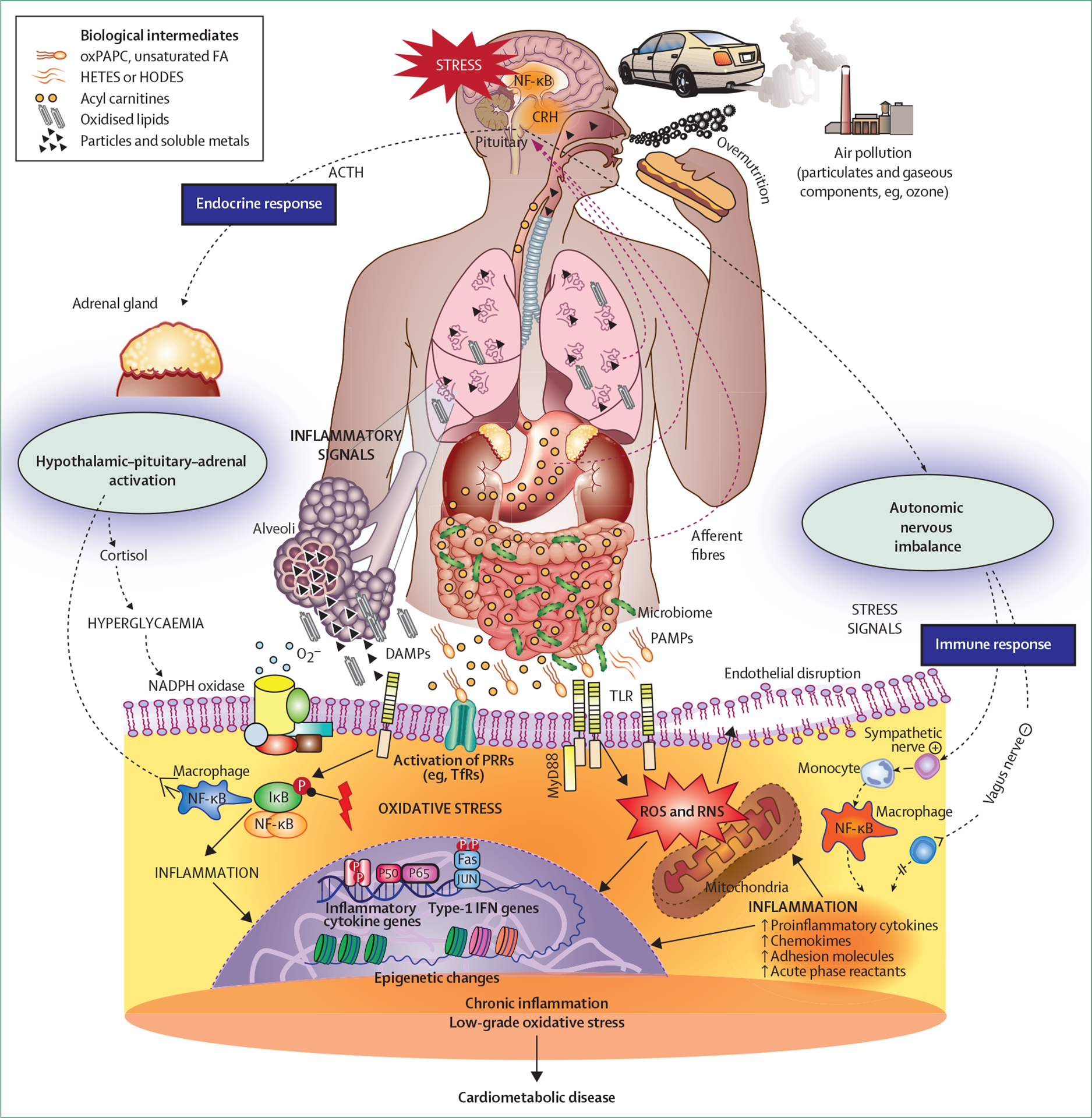
Mechanisms of PM_2·5_-mediated metabolic and cardiovascular effects ACTH=adrenocorticotropic hormone. AMP=activated molecular patterns. CRH=corticotropin releasing hormone. DAMPs=disease-activated molecular patterns. FA=fatty acids. HETES=hydroxyeicosatetraenoic acids. HODES=hydroxyoctadecadienoates. IFN=interferon. IκB=IκB kinase. MyD88=myeloid differentiation primary response protein 88. NADPH=nicotinamide adenine dinucleotide phosphate. NF-κB=nuclear factor κB. oxPAPC=oxidised phospholipid 1-palmitoyl-2-arachidonoyl-sn-glycero-3-phosphorylcholine. PAMPs=pathogen. PRRs=pattern recognition receptors. RNS=reactive nitrogen species. ROS=reactive oxygen species. TfR=transferrin receptor. TLR=toll-like receptors.

Understanding of the pathways by which air pollution might contribute to the risk of heart and metabolic problems, while still evolving, have been refined over the past decade.^5^ The generally understood pathways by which air pollution might potentiate cardiometabolic disease are depicted in figure 2. Conceptually, these pathways could be viewed as follows: primary initiating pathways that are localised in the airways and lungs, and might comprise oxidative stress, ion channel or receptor activation, and inflammation; systemic transduction of metabolic effects through release of biological intermediates (eg, oxidised lipids, cytokines, activated immune cells, microparticles, microRNA, endothelins); autonomic imbalance; hypothalamic–pituitary–axis activation; nanoparticles or pollutants reaching the circulation or transmitted via neurological pathways; and effector mechanisms in insulin responsive organs. These pathways could include one or multiple mechanisms, such as endothelial barrier disruption; adipose and hepatic inflammation; endoplasmic reticulum stress; brown adipose tissue and mitochondrial dysfunction; end-organ responses to autonomic imbalance and CNS activation, including hypothalamic inflammation or dysfunction, and hypothalamic–pituitary–axis activation (eg, vasoconstriction, increased blood pressure, and hypercortisolaemia); and organ responses to circadian disruption.

**Figure 3:**
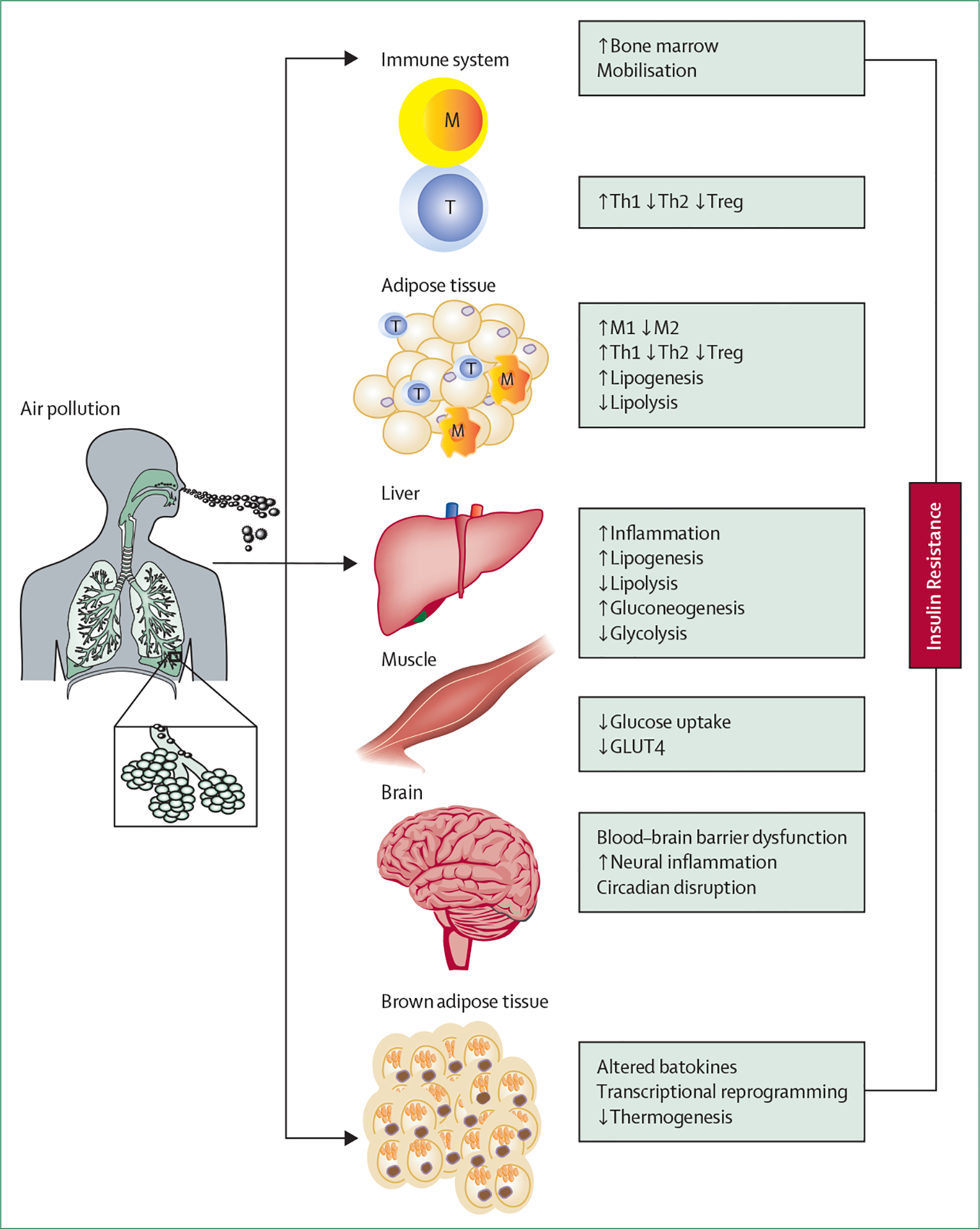
Organ-specific mechanisms of air pollution-related insulin resistance GLUT4=glucose transporter type 4. M=macrophages. T=T cells. Th1=type 1 helper T cell. Th2=type 2 helper T cell. Treg=regulatory T cell.

Specific pathways relevant to insulin resistance are depicted in figure 3. In reality, the pathways can be much more complex and mutually dependent than what is conceptualised in figure 2 and figure 3, with temporally overlapping timescales. Also, biological effects might vary based on individual predilection and the composition of air pollution.

### Primary initiating pathways

Oxidative stress is the first hierarchical response in reaction to air pollution exposure, occurring initially in the respiratory tract and lungs, and playing a key role in many of the subsequent pathways.^57^ Chemical transformation of endogenous thiols, fatty acids, and lipids—resulting in the additional generation of reactive products—can occur, and has been implicated in translating systemic responses.^58^ This transformation is evidenced by markers and mediators such as 8-iso-prostaglandin2α, malondialdehyde, oxidised-LDL, oxidised HDL species, and lipid peroxidation biomarkers, which have all been noted to be associated with higher levels of PM_2·5_ and other air pollutant exposure in humans.^58–60^ Conversely, in reducing air pollution levels with filtration and treatment with antioxidants, fish oils have been shown to mitigate markers of oxidant stress in response to PM exposure.^61^

### Systemic transduction of air pollution effects

The direct translocation of pollutant particles and other leachable components is now well established in humans.^3,5,10^ The formation and transmission of reactive biological intermediates, including oxidant stress-generated signal transduction molecules, might mediate systemic metabolic effects.^10^ Oxidative stress produced in the lung is important in transducing systemic insulin resistance and inflammation. Inhalational exposure to concentrated PM_2·5_ for 10 days in mice has been shown to increase circulating saturated fatty acids (eg, palmitate), unsaturated fatty acids (eg, palmitoleate and myristoleate), acyl carnitines, and bile acid metabolites. Overexpression of extracellular superoxide dismutase in the lung prevented not only the increase in circulating biomarkers, but vascular inflammation in response to concentrated PM_2·5_ exposure.^62^ A 2-week exposure to ultrafine particles (diesel exhaust) led to increased lipid peroxidation and reduced plasma HDL protective capacities. Malondialdehyde and products of arachidonate metabolism, such as hydroxyeicosatetraenoic acids and hydroxyoctadecadienoates, occurred preferentially in the plasma and liver, and less so in the lung.^63^ In a double-blind, randomised trial in China, air filtration lowered participants’ blood pressure and various metabolic mediators, including inflammatory and oxidant stress measures.^64^ The effect of inflammation resolution measures in mitigating responses to PM exposure needs to be better understood.

Air pollution components or biological intermediates can be sensed by multiple families of sensing receptors, including toll-like receptors (TLR2 and TLR4), nucleotide-binding domain leucine-rich repeats of nucleotide oligomerisation domain-like receptors, and transient receptor potential (TRPA1 and TRPV1) channels.^10,57^ A few human panel studies and double-blind interventional trials of air-purifiers have shown an association between PM_2·5_ exposure and several plasma inflammatory markers (eg, C-reactive protein, IL-6, and tumour necrosis factor-α). Reduction in some of these markers with air filtration that suggests a cause and effect relationship, but the results of these studies have not always been consistent.^61,64–66^

Experimental studies with chronic inhalation exposure to concentrated ambient PM_2·5_ have implicated infiltration of monocytes as a cause of inflammation and an M1–M2 switch in adipose tissue. TLR4 appears to be crucial in the effects of PM_2·5_, including haematopoietic efflux and tissue infiltration.^67^ C–C chemokine receptor type 2 (CCR2) and chemokine (C–X–C motif) receptor 3 also appear to be crucial in the inflammatory response to PM_2·5_ through distinct monocyte and T-cell mediated pathways.^68^ *CCR2*-knockout mice, fed high-fat chow, show reduced adipose inflammation, improved whole-body insulin resistance, and reduced hepatic lipid accumulation.^69^ Chemokine (C–X–C motif) receptor 3 knockout (*CXCR3*) plays a role in the migration of activated T-cell populations (CD44^+^, CD62L^−^, and CD4^+^) into adipose tissue and lungs.^68^ The overall effects of chronic exposure to concentrated airborne particulate matter in the animal model appears to be consistent with systemic type 1 immunity (type 1 helper T cells [Th1]) dominant immune responses, with a skew from M2 towards M1 and from Th2 towards Th1 in tissue depots.

Dysregulation of the autonomic nervous system and sympathetic activation have been noted acutely in humans and animal models with inhalational exposure to air pollution.^58,70,71^ Given the well known relationship between sympathetic activation and insulin resistance, activation of this pathway in response to PM_2·5_ and ultrafine particle exposure (commonly inferred by an acute reduction in heart rate variability or increase in blood pressure and elevation in catecholamines) in both panel studies and with direct inhalational exposure in humans is supportive of the relevance of this pathway in insulin resistance with air pollution exposure.^72^ Randomised filtration interventions to lower air pollution levels also provide supportive evidence for the causal involvement of sympathetic nervous system and hypothalamic–pituitary–adrenal axis activation.^64,73^ Acute experiments in canine models and studies in chronically cannulated mice exposed to concentrated ambient PM_2·5_ have documented increased blood pressure, with evidence of CNS and sympathetic nervous system activation in response to PM_2·5_ exposure.^74,75^ Abnormalities in autonomic function or blood pressure responses are, however, not consistently observed, and might relate to compositional differences of pollution exposures and study design.^76,77^ Inhalation of diesel exhaust ultrafine particles caused a significant elevation within 30 min in muscle sympathetic nerve activity metrics compared with filtered air. Heart rate significantly increased, and there were trends for systolic and diastolic blood pressure elevations, probably haemodynamic consequences of sympathetic nervous system activation. Other components of air pollution, such as volatile organic compounds (eg, acrolein and 1,3-butadiene), might contribute to elevated blood pressure in participants with increased sympathetic tone.^78^

### Effector mechanisms in insulin-responsive organs

Previous reviews have extensively examined the effect of air pollution on conduit artery and resistance vessel function.^58,70^ Alterations in endothelial function might translate into muscle insulin resistance and hypertension. Endothelial barrier dysfunction has been noted in sites such as the blood–brain barrier in response to PM_2·5_ and ultrafine particle exposure. Endothelial barrier dysfunction might provide a potential mechanism by which PM_2·5_ can predispose individuals to peripheral effects that include brown adipose tissue dysfunction, white adipose tissue inflammation, and insulin resistance.^79,80^

### Adipose and hepatic inflammation

Inhalational exposure to PM_2·5_ hastens the development of insulin resistance and abnormal glucose tolerance in both diet-induced and genetic models of obesity.^80^ Genetic ablation of *CCR2* and neutrophil cytosol factor 1 (*p47phox*) improves concentrated airborne particulate-induced insulin resistance and adipose inflammation, and is indeed consistent with the important role of CCR2 and oxidant stress pathways in inflammatory monocyte and macrophage recruitment into adipose tissue and insulin resistance.^69,81^ Chronic concentrated ambient PM_2·5_ exposure has been associated with a multitude of abnormalities in the liver involving glucose transport, metabolism, lipolysis–lipogenesis balance, altered insulin signalling, inflammation, and findings consistent with steatohepatitis.^69,82,83^

### Endoplasmic reticulum stress

Experimental studies provide substantial evidence that supports activation of all three components of the evolutionarily conserved unfolded protein response or endoplasmic reticulum stress with PM_2·5_ exposure.^84,85^ Endoplasmic reticulum stress is an essential regulator of adipocyte lipid metabolism and adipose tissue inflammation. Concentrated airborne particulate exposure in *C57BL*/6J mice for 10 months resulted in increased adipocyte size and lipid deposition in white adipose tissue, together with increased endoplasmic reticulum stress genes (such as *BiP*/*GRP78*, *Xbp-1*, and *Edem1*) in white adipose tissue, along with genes involved in lipogenesis (*Acaca*), transport (*CD36*), triglyceride synthesis (*Dgat2*), and adipocyte differentiation or lipid droplet formation (*Smaf1*, *Ceacam1*, *Fsp27*, *Plin1*, and *Fit2*).^86^

### Brown adipose tissue effects

In experiments performed in metabolic cages in *C57BL*/6J mice, concentrated airborne particulate exposure resulted in reduced VO_2_ and VCO_2_ levels and decreased thermogenesis, consistent with a reduction in metabolism.^71,87,88^ These effects were associated with a reduction in uncoupled protein-1 expression in brown adipose tissue and dysregulation of multiple transcriptional regulators of brown adipose tissue metabolism and function.^88^

### CNS inflammation in key energy regulatory centres

A considerable amount of human data suggest that hypothalamic inflammation and dysregulation of energy balance and glucose homoeostasis might be at the core of type 2 diabetes.^89^ Both short-term and long-term exposure to air pollutants have been shown to induce hypothalamic inflammation and reduce metabolism, with reversal noted in response to inhibition of inflammatory mediators in the brain.^88,90^ Energy balance is also impacted through the aforementioned brown adipose tissue mechanisms and through alterations in the hypothalamic–pituitary–adrenal axis. Increased secretion of corticosterone and catecholamines has been demonstrated in animal models and humans in response to both PM_2·5_ and ozone exposure.^64,71,90–92^ In a post-hoc analysis of the MESA cohort, higher levels of annual NO_2_ and PM_2·5_ were associated with higher epinephrine and dopamine levels.^93^

### Circadian disruption

Previous experimental studies have demonstrated that circadian genes are some of the most commonly expressed genes in response to air pollution exposure.^94^ *C57BL*/6 mice exposed to concentrated ambient PM_2·5_ showed insulin resistance, reduced energy expenditure, decreased thermogenesis, decreased metabolism, and dysregulated circadian genes that reversed with cessation of exposure.^94^ A study comparing exposure to PM_2·5_ with light at night exposure showed that, although PM_2·5_ and light at night induced an identical phenotype of insulin resistance and metabolic dysfunction, the transcriptional and epigenetic pathways (including differentially expressed circadian genes) seemed to differ. In humans, metabolic syndrome parameters, sleep deprivation, and depression (circadian syndrome) were highly correlated with PM_2·5_ exposure.^71^

### Epigenomic changes

Epigenetic reprogramming in response to environmental exposures might represent a crucial buffer against an adverse health response by regulating gene expression and chromosome integrity.^95^ Chronic PM_2·5_ exposure promoted substantial chromatin remodelling, especially at promoter and enhancer sites that were pliable, with cessation of exposure resulting in a reversal of changes in chromatin accessibility and of expression of transcripts—notably those involved in insulin action, circadian rhythm, and inflammation.^71^ Methylation changes and epigenomic changes in networks enriched for pathways related to inflammation, thrombosis, insulin resistance, and lipid metabolism have all been noted.^95^

## Opportunities for mitigating air pollution cardiometabolic health effects

One of the most important first steps in preventing air pollution-mediated metabolic diseases worldwide is to acknowledge the impact of environmental factors such as air pollution on these conditions. Studies on pollution inequity or the differences between environmental health exposures have shown that disproportionally high direct exposure to pollution and climate-related risk factors results in substantial life expectancy disparities, even between adjacent post-codes.^44,96^ There is a direct link between climate change and air pollution, and both can have direct and indirect effects on health.^97^ Overall, climate change might worsen air quality by altering meteorological air pollution removal processes and intensifying responses in atmospheric chemistry from both man-made and natural sources.^98^ Although strategies to reduce climate change can have co-benefits in reducing air pollution, there might also be short-term unintended responses that could affect climate. For instance, a reduction of sulphate emissions from ships has been linked to reduced cloud cover and global warming.^99,100^ There is a real need for dissemination of the health impacts of climate, and dire need for integrating the health impact of climate in all policy decisions.

**Figure 4:**
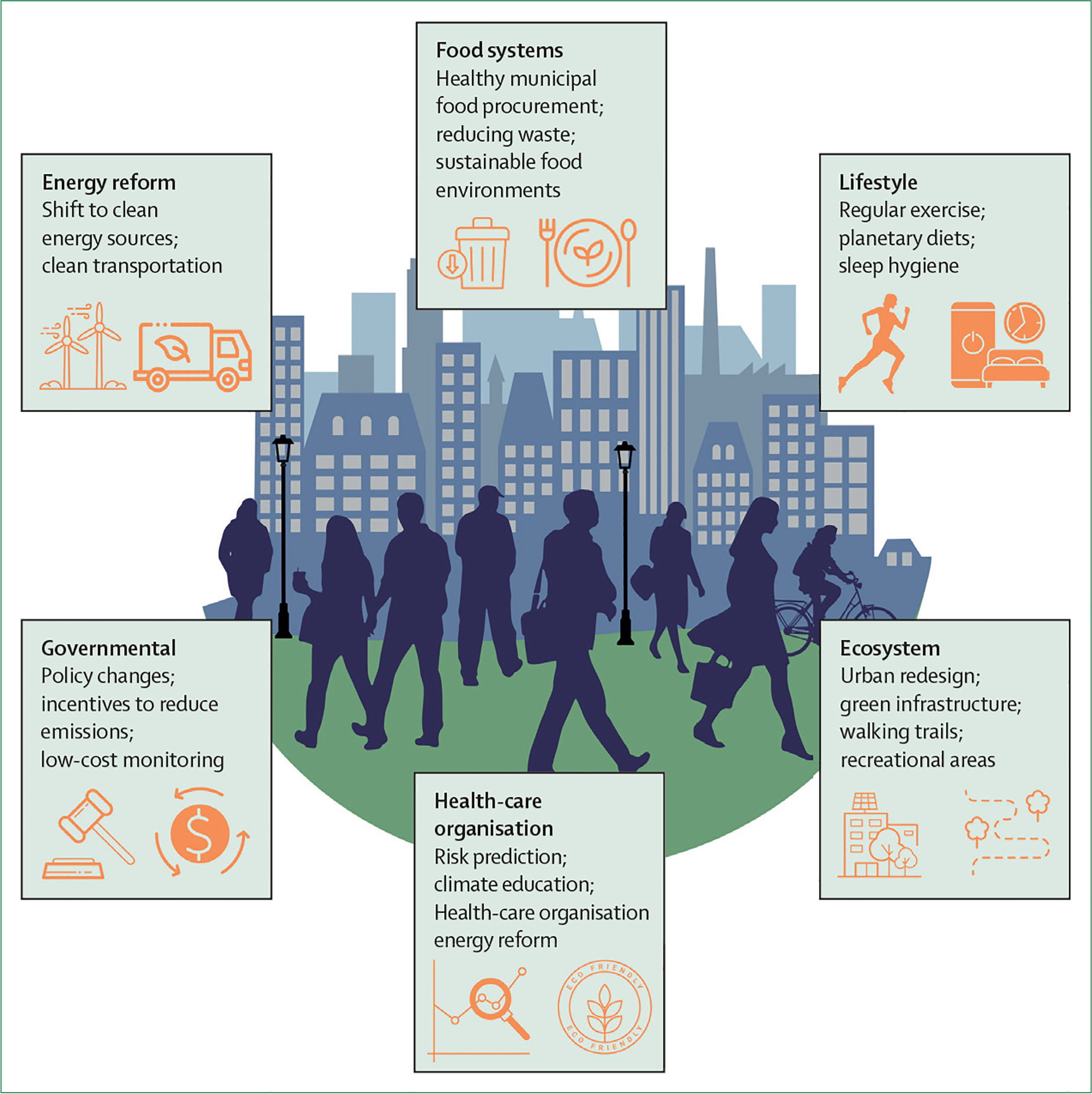
Mitigation strategies to control air pollution-related cardiometabolic disease

Integrated mitigation strategies to address cardiometabolic health that might help improve physical activity and healthy nutrition, but also curb air pollution, ultimately advancing health, climate, and equity goals, are illustrated in figure 4. Policies that endorse clean energy transition, enforcement of pollution control standards, and incentives for reducing processed foods and increasing consumption of plant-based foods, will substantially reduce the burden of type 2 diabetes.^3,101^ The simultaneous promotion of clean energy modes of public transportation and active transport, such as walking and biking, can help reduce air pollution and improve physical activity. Health impact studies indicate that the health and economic benefits of increasing active transportation simultaneously with clean energy transportation vastly exceed those divined by policies promoting electric mobility transition alone.^102^ Thus, increasing active transport must be integral to clean energy policy, and new innovative urban design and planning, such as the 15-minute City in Paris, superblocks in Barcelona, and car-free neighbourhoods in Freiburg, are greatly needed.^103^ Similarly, a shift to locally sourced plant-based diets, even partly, might considerably reduce greenhouse gas emissions and PM_2·5_ pollution, and improve metabolic health.^104^ Urban redesign with low-carbon sustainable construction materials, green infrastructure, and walking trails might help increase physical activity while reducing embodied carbon and air pollution from the manufacturing, transportation, installation, and disposal of building materials.^104^

The American Heart Association statement on personal protective actions against air pollution, which considers both exposure risks and individual susceptibility, provides a useful framework for individuals at high risk for air pollution-mediated health effects.^61^ Portable air cleaners are practical and inexpensive in-home strategies suited for at-risk populations, and can help acutely reduce PM_2·5_ exposures by as much as 60%.^61^ Several small, short-term, randomised studies in humans have provided proof of concept that reductions in PM_2·5_ exposures with portable air cleaners can result in rapid, albeit small, reductions in blood pressure and other markers of adverse cardiometabolic risk effects, including inflammation (eg, lower C-reactive protein and IL-6), which are well established risk factors for type 2 diabetes.^61^ In areas facing very high levels of PM_2·5_, or during extreme air pollution events (such as occurs regionally for days to weeks from wildfires), indoor portable air cleaners with high-efficiency particulate air filtration, in addition to N95 face masks when travelling outdoors, might be warranted for individuals at high risk of cardiometabolic effects. Some small studies have shown the potential for health protection from wearing face masks; however, more work is needed in this regard.^21,61^

Health-care organisations and non-profit organisations are well positioned to not only urge governments to take action to prevent pollution-related cardiovascular disease by setting targets and timetables for pollution reduction, but to lead the way in attaining net zero emission targets. The economic impact of the pollution derived from the US health-care system is comparable to the economic impact attributable to medical errors.^105^ Importantly, the economic impact of pollution is primarily driven by air pollution.^105^ By mandating sustainability standards in hospitals and health-care organisations, an immediate impact on emissions can be seen, together with cost savings. Including planetary diets in hospitals will allow much-needed alignment of health benefits with planetary goals.^106^

### Air pollution and exercise

Acute elevations in air pollution might discourage physical activity and increase sedentary behaviour in adults and children. Several regulatory authorities, such as the UK Department of Environment and the US Environmental Protection Agency, recommend that the general population should restrict outdoor physical activity on days when PM_2·5_ levels exceed predefined thresholds. Based on health impact modelling studies, even in areas with very high PM_2·5_ concentrations (annual average 120 μg/m^3^), the overall favourable effects of outdoor exercise—either in the form of cycling or walking—obviates the negative effects of inhaling high levels of air pollution, with a reduction in mortality risk with cycling of 15% in Beijing and 14% in New Delhi, assuming annual average PM_2·5_ concentrations of 95 μg/m^3^ in Beijing and 120 μg/m^3^ in New Delhi.^107^ It is important to note that these reported reductions in mortality risk are in healthy individuals, and whether these results are applicable to high-risk individuals (eg, individuals with type 2 diabetes and cardiovascular or renal disease) is unknown. Overall, the health benefits of regular exercise appear to outweigh the risks of pollution exposure in all but the most extreme settings. Nevertheless, it is a reasonable precautionary approach to exercise indoors or outdoors away from pollution sources (eg, roadways) or postpone the timing of exercise, if possible, to less polluted periods.

## Challenges and opportunities for the future: air pollution and beyond

Given the importance of air pollution as a relevant risk factor for cardiometabolic disease, progressive reduction in fossil fuel emissions through the transition to clean energy is expected to decrease the incidence of cardiometabolic disease. Although air pollution emissions are decreasing, worsening trends in physical activity, diet, and exposures to chemical pollution could outweigh these improvements.

The creation of sustainable environments with attention to urban spatial design to encourage walking and active transportation, and with attention to the key provisioning systems of food supply, energy, transportation, buildings, sanitation, water, and green infrastructure, provide the greatest opportunity to reduce cardiometabolic disease while ensuring climate and equity goals. Currently, inadequate and antiquated provisioning systems are responsible for multiple adverse health exposures, such as regional air pollution, local soil or water pollution, adverse nutritional exposures, and climate risks. Policy, appropriate governance, and legal frameworks enable execution of urban planning decisions and regulation of risk exposures. The shortage of policy levers and governance of urban decisions is at the core of unregulated urban proliferation and acceleration of adverse cardiometabolic outcomes in many urban environments worldwide. The economic valuation of the negative externalities of air pollution, including its impact on health, can help justify clean energy initiatives with rapid recoupment of initial investments. The Organization for Economic Co-operation and Development, using such a model, estimated that global air pollution-related health-care costs will increase from US$21 billion in 2015 to $176 billion in 2060.^108^ Furthermore, the market effects of outdoor air pollution, due to impacts on labour productivity, health expenditure, and agricultural crop yields, might reach 1% of the global gross domestic product by 2060. Importantly, air pollution continues to be a negative externality that is not paid for by either the consumer or the polluter. The use of financial instruments to regulate pollution (polluter pays) could help catalyse a shift to cleaner solutions.

Although global PM_2·5_ guidelines are important for alignment between country policies, and a step in the right direction, national, state, and local strategies must also be identified and developed to reduce PM_2·5_. Sector-specific plans might also help with optimising the cost-effectiveness of PM_2·5_ interventions and improving industry accountability.^108^

A revolution in personal devices that quantify human health and provide exposure information to a range of chemicals and entities, in addition to PM_2·5_, provides an opportunity to develop better predictors of human health. Air pollution coexists with enduring elements in the built environment and other key provisioning systems that foster sedentary habits, poor nutritional choices, and exposures to chemicals implicated in type 2 diabetes. The exposome concept strives to capture the diversity and range of internal and external exposures in the environment, such as pollution, synthetic chemicals, dietary constituents, psychosocial stressors, and climate related factors, and their corresponding biological responses. Technological advances, such as unbiased high-throughput genomic and epigenomic approaches, high-resolution metabolomics, and network science, have allowed us to take the first steps toward a comprehensive assessment of the exposome and its effects on cardiometabolic health.^109,110^ Linking exposomic signatures with biological surrogates and endpoints might provide new insights into the pathophysiology of cardiometabolic disease.^111^

## Research gaps

There are still substantial research gaps in our knowledge of air pollution and its effect on cardiometabolic disease. A greater mechanistic understanding of the impact of the specific subcomponents of PM_2·5_ is required. Furthermore, interventional studies evaluating the effect of PM_2·5_ mitigation approaches, such as personal air filtration devices, and the effect of PM_2·5_ mitigation approaches on cardiometabolic outcomes, could provide valuable evidence for development of individual-level interventions. The effect of air pollution needs to be understood in the context of multiple social and environmental exposures that might co-segregate with air pollution and drive risk associations with cardiometabolic health. Given the increased recognition of the dominant role that non-genetic factors play in type 2 diabetes, an effort to characterise the exposome at a scale similar to that of the human genome is warranted.

## Conclusion

Air pollution is a major determinant of cardiometabolic disease globally, and a major contributor to climate change. Efforts to reduce air pollution exposure and mitigate its health effects involve raising individual awareness, appropriate policy levers to reduce sources of air pollution, and measures to reduce personal health impact.

## Supplementary Material

Supplementary Material.Air pollution exposure and cardiometabolic risk. Lancet Diabetes Endocrinol.

## Search strategy and selection criteria

References for this Review were identified through searches of PubMed for articles published from Jan 01, 2006, to June 30, 2023, using the search terms “diabetes”, “insulin resistance”, “exercise“, “obesity”, “cardiovascular disease”, “health impact”, and “cardiometabolic”, in combination with the term “air pollution”. Articles resulting from these searches, and relevant references cited in those articles, were reviewed and included when appropriate. Articles were required to have a full-text English version. Both human and animal studies were considered. No additional search restriction or selection criteria was applied.
