# Supplementary material for: Air pollution exposure and cardiometabolic risk
Source: Lancet Diabetes Endocrinol. Author manuscript; Available in PMC 2024 Jul 23. (PMC11264310; doi:10.1016/S2213-8587(23)00361-3)
Supplement: Supplementary Material.Air pollution exposure and cardiometabolic risk. Lancet Diabetes Endocrinol. [file NIHMS2008326-supplement-Supplementary_Material_Air_pollution_exposure_and_cardiometabolic_risk__Lancet_Diabetes_Endocrinol_.pdf]

THE LANCET
Diabetes & Endocrinology

Supplementary appendix

This appendix formed part of the original submission and has been peer reviewed. We post it as supplied by the authors.

Supplement to: Rajagopalan S, Brook RD, Salerno PRVO. Air pollution exposure and cardiometabolic risk. *Lancet Diabetes Endocrinol* 2024; published online Feb 1. https://doi.org/10.1016/S2213-8587(23)00361-3.

Appendix

Contents:

- **Appendix Table 1: Air Pollution and Insulin Resistance. Page 2 and 3**
- **Appendix Table 2: Air Pollution and Diabetes incidence, mortality and obesity. Page 4 to 9**
- **References. Page 10 to 12.**

APPENDIX TABLES

Appendix Table 1: Air Pollution and Insulin Resistance

Author	Location	Pollutants	Number of Subjects	Study Design	Findings	Ref.
Brook (2013)	Michigan, USA	$PM_{2.5}$	25	Intervention time series study. Participants from rural Michigan were transported to urban city (exposure block) and were observed for 5 days. Insulin resistance, HRV, inflammatory factors, vascular function were measured before, during and after exposure	Each 10 μg/m3 increase in sub-acute $PM_{2.5}$exposures was associated with increased HOMA-IR (+0.7, 95% CI: 0.1, 1.3) and reduced HRV (SDNN -13.1ms, 95% CI: -25.3, -0.9). No alterations in other outcomes (inflammatory markers, vascular function) occurred in relation to $PM_{2.5}$ exposures	1
Brook (2016)	Beijing, China	$PM_{2.5}$<br>BC	65	Panel study. Participants had 4 repeated visits across 4 seasons. Daily ambient $PM_{2.5}$ and BC exposure were assigned using personal monitors. BP and insulin resistance were measured.	$PM_{2.5}$ exposure (1 to 7 days prior) were significantly associated with SBP (2.0-2.7 mm Hg per 67.2 μg/m3 increase in PM), and cumulative BC exposure (2 to 5 days prior) were associated with DBP (1.3-1.7 mmHg per 3.6 μg/m3). BC and PM (lag days 4 and 5) were associated with worsening insulin resistance (BC: 0.18-0.22 unit increase per SD of BC and 0.18-0.22 unit increase per SD of PM)	2
Chen (2016)	California, USA	NO_2<br>O_3<br>$PM_{2.5}$	1,023	Cross-sectional cohort study of 1,023 Mexican-Americans selected from the BetaGene study. Participants underwent oral and intravenous glucose tolerance, and ambient air pollution was assigned using local monitors.	Short-term $PM_{2.5}$ exposure was associated with lower insulin sensitivity and HDL-to-LDL cholesterol ratio and higher fasting glucose and insulin, HOMA-IR, total cholesterol, and LDL cholesterol (all $P \leq 0.036$). Long-term $PM_{2.5}$ exposure (annual average $PM_{2.5}$) was associated with higher fasting glucose, HOMA-IR, and LDL ($P \leq 0.043$)	3
Thiering (2016)	Munich & Wesel, Germany	PM_{10}<br>NO_2<br>$PM_{2.5}$	397	Two prospective birth cohort studies consisting of a total 397 child participants without diabetes in Munich and Wesel, Germany. Glucose and fasting insulin were measured.	HOMA-IR increased by 17.0% (95% CI: 5.0, 30.3) and 18.7% (95% CI: 2.9, 36.9) for every 2 SD increase in NO_2 (10.6 μg/m3) and PM_{10} (6 μg/m3) respectively.	4

Wolf (2016)	Augsburg, Germany	PM_{10} NO_2	2,944	Cross-sectional study of participants of the KORA (Cooperative Health Research in the Region Augsburg) F4 study conducted in southern Germany (2006-2008). Fasting HOMA-IR, glucose, insulin, HbA1c, leptin, and high-sensitivity C-reactive protein were measured. PM_{10} was assigned using land-use regression models.	Each 7.9 μg/m3 increment in PM_{10} was associated with a 15.6% increase in HOMA-IR and 14.5% increase in insulin. NO_2 dioxide was also associated with HOMA-IR, glucose, insulin, and leptin. Prediabetes accentuated the relationship between pollutants and insulin resistance. No association was seen for HbA1c level	5
Khafaie (2017)	Pune, India	PM_{10}	1,213	Cross-sectional study of 1213 patients with diabetes living in Pune, India. PM_{10} was assigned from the dispersion model. Fasting plasma glucose, HbA1c, 2 h post meal plasma glucose (2hPG), HOMA-IR, HOMA-β and disposition index (DI) were measured	1 SD increment in background concentration of PM_{10} at residential places (43.83 μg/m3) was significantly associated with 2.25 mmol/mol increase in HbA1c, 0.38 mmol/l increase in 2hPG, 4.89% increase HOMA-IR.	6
Dang (2018)	Germany, USA, and Belgium	PM_{10} NO_2 $PM_{2.5}$	11,656	Meta-analysis of six cohort studies conducted in Germany, USA, and Belgium. HOMA-IR, fasting plasma glucose, insulin, HbA1c, and leptin levels were examined.	A 1 μg/m3 increase in NO_2 was associated with pooled effect changes of 1.25% in HOMA-IR (95% CI: 0.67, 1.84) and 0.60% in insulin (95% CI: 0.17, 1.03). A 1 μg/m3 increase in PM_{10} was associated with pooled effect changes of 2.77% in HOMA-IR (95% CI: 0.67, 4.87) and 2.75% in insulin (95% CI: 0.45, 5.04). No association was seen for $PM_{2.5}$	7
Zhang (2021)	Augsburg, Germany	$PM_{2.5}$ NO_2 O_3	4,261	Longitudinal cohort study using data from the KORA S4, F4, and FF4 examinations.	HOMA-IR significantly increased by 2.5-3.5% for PM_{10},$PM_{2.5}$ and NO. The rate of change of HOMA-IR over multiple repeat visits was positively correlated with $PM_{2.5}$ levels	8
Zhan (2021)	Nanjing, China	$PM_{2.5}$	47,471	Cross-sectional study of 47,471 patients who participated in physical examinations in Eastern China. Fasting plasma glucose was measured.	FBG increased by 0.0030, 0.0233, and 0.0325 mmol/L at lag 0–7, 0–21, and 0–28 days after a 10 μg/m3 increase in $PM_{2.5}$.	9

Abbreviations: CI: confidence interval; BC: black carbon; HOMA-IR: Homeostatic Model Assessment of Insulin Resistance; HRV: heart rate variability; SDNN: standard deviation of normal-to-normal R-R interval; HDL: High-Density Lipoprotein; LDL: low-density lipoprotein; BP: blood pressure; SBP: systolic blood pressure; DBP: diastolic blood pressure; DI: disposition index; 2hPG: 2-hour post-meal plasma glucose

Appendix Table 2: Air Pollution and Diabetes incidence, mortality and obesity.

Author (Year)	Location	Pollutants	Number of Subjects	Study Design	Findings	Ref.
**Diabetes Incidence**						
Eze (2015)	Europe & North America	$PM_{2.5}$<br>NO_2	241,835	Large meta-analysis comprising 13 total studies, including longitudinal, cross-sectional, case-control, and ecologic. Incident T2DM was measured and risk ratios were calculated.	Exposure to $PM_{2.5}$ was associated with a pooled relative T2DM risk of RR = 1.10 (95% CI: 1.02, 1.18). Exposure to NO_2 was associated with a pooled relative T2DM risk of RR = 1.08 (95% CI: 1.00, 1.17)	10
Kutlar Joss (2023)	Europe, North America, China, Australia	NO_2<br>$PM_{2.5}$<br>PM_{10}<br>EC	6,222,440	Meta-analysis consisting of 11 cohort studies and 10 cross-sectional studies.	NO_2 exposure was associated with higher diabetes prevalence (RR 1.09; 95% CI: 1.02; 1.17 per 10 µg/m3). This association was less pronounced for diabetes incidence (RR 1.04; 95% CI: 0.96; 1.13 per 10 µg/m3). Associations for $PM_{2.5}$, PM_{10}, and EC were positive but less precise and had less studies.	11
Sorensen (2023)	Denmark	NO_2<br>EC<br>UFP<br>$PM_{2.5}$	2,757,813	Cohort study including all Denmark residents of age 35-80. Included sociodemographic variables, financial stress, population density, road traffic noise, and green space in association analysis.	$PM_{2.5}$ exposure was associated with T2DM risk of HR = 1.17 (95% CI: 1.13, 1.21) per 5 µg/m3. UFP exposure was also associated with T2DM, with HR = 1.16 (95% CI: 1.13, 1.19) per 10,000 UFP/cm3. EC was associated with HR = 1.10 (95% CI: 1.08, 1.12). NO_2 was associated with HR = 1.10 (95% CI: 1.08, 1.11).	12
Liang (2023)	USA<br>China<br>Japan<br>Australia<br>Denmark	$PM_{2.5}$<br>PM_{10}<br>NO_2<br>SO2	4,868,627	Meta-analysis of 31 studies. Measured gestational diabetes mellitus (GDM)	Of all pollutants, observed the strongest relationship between $PM_{2.5}$ exposure and GDM risk, RR = 1.076 (95% CI: 1.032, 1.123) per 10 µg/m3 $PM_{2.5}$ during preconception, and RR = 1.135 (95% CI=1.061–1.215) during the first two trimesters. Associations between GDM and PM_{10}, NO_2, and SO2 were significant but modest.	13
**Obesity**						
Matthiessen (2018)	Germany	NO_2<br>PM_{10}	4457	Prospective population-based cohort study located in three	NO_2 was positively associated with Metabolic Syndrome prevalence, OR	14

		$PM_{2.5}$		adjacent cities within the highly urbanized German Ruhr Area.	increase per IQR of 1.12 (95%-CI 1.02–1.24, IQR = 6.1 µg/m3). PM_{10} and $PM_{2.5}$ positively associated with Metabolic Syndrome incidence, with ORs of 1.14 (95%-CI 0.99–1.32, IQR = 2.1 µg/m3) and 1.19 (95% CI: 0.98,1.44, IQR = 1.5 µg/m3) per IQR, respectively.	
Lee (2019)	South Korea	$PM_{2.5}$	119,998	Cohort study with participants who received health screening provided by the Korean Medical Insurance Corporation in a National Health Insurance Service-National Health Screening Cohort (NHIS-HEALS) between 2009 and 2010.	A 10-µg/m3 increase in 1-year average $PM_{2.5}$ level was significantly associated with a 7% increased risk for developing metabolic syndrome (HR = 1.070, 95% CI: 1.032, 1.110), 1% increased risk of waist based obesity (HR = 1.510, 95% CI: 1.422,1.601), a 49% increased risk of hypertension obesity (HR = 1.499, 95% CI: 1.441,1.559), a 46% increased risk of hypertriglyceridemia (HR = 1.468, 95% CI: 1.424,1.513), a 62% increased risk of low HDL-C (HR = 1.627, 95% CI: 1.564,1.693), and a 38% increased risk of hyperglycemia (HR = 1.380, 95% CI: 1.338,1.423).	15
Kim (2019)	USA	NO_2<br>O_3<br>$PM_{2.5}$	158	Subcohort of kindergarten and first grade children were recruited from public schools across Southern California communities and followed through their high school years.	A 1 SD change in long-term NO_2 exposure was associated with a 11.3 mg/dL higher level of total cholesterol (p=0.04) and 9.4 mg/dL higher LDL (p=0.04). Among obese participants, associations between long-term NO_2 and total cholesterol and LDL-cholesterol were 4.5 and 9 times larger than the associations in non-obese participants (pinteraction=0.008 and 0.03, respectively). Increased short-term O_3 exposure was associated with higher triglyceride and VLDL levels (p=0.04), lower HDL cholesterol levels (p=0.03), and higher hepatic fat levels (p=0.02). Long-term$PM_{2.5}$ exposure was associated with higher levels of insulin area under the curve (p=0.03). No further significant associations with short- or long-term air pollutants and BMI, other	16

					measures of adiposity, and cardiometabolic outcomes were found.	
Li (2021)	United Kingdom	$PM_{2.5}$ $PM_{2.5-10}$ NO_2 NO	449,006	Cohort study in the UK biobank consisting of participants without T2DM at baseline.	Air pollution score was significantly associated with a higher risk of T2DM. Observed a significant interaction between the air pollution score and obesity on T2DM risk. Genetic risk score for T2DM or obesity did not modify the relationship between air pollution and T2DM risk	17
Voss (2021)	Netherlands Norway	Noise	144,082	Analysis that included two european cohorts, HUNT, from Norway, and Lifelines from the Netherlands.	An 1 IQR higher day-time noise (5.1 dB(A)) was associated with 1.1% (95% CI: 0.02, 2.2) higher high-sensitivity C-reactive protein (hsCRP), 0.7% (95% CI: 0.3, 1.1) higher triglycerides, and 0.5% (95% CI: 0.3, 0.7) higher HDL; only HDL's remained significant after adjusting for air pollution.<br><br>PM_{10} (2.0 µg/m3) and NO_2 (7.4 µg/m3) were associated with higher triglycerides (1.9%, 95% CI: 1.5, 2.4 and 2.2%, 95% CI: 1.6, 2.7), independent of adjustment for noise. NO_2,was also associated with hsCRP (1.9%, 95% CI: 0.5, 3.3).	18
Bowe (2021)	USA	$PM_{2.5}$	3,902,440	Cohort of US Veterans built from the multiple VA databases	A 10-µg/m3 increase in $PM_{2.5}$ concentration was associated with an increased risk of incident adult obesity (HR = 1.08, 95% CI: 1.06, 1.11) and weight (HR = 1.07, 95% CI: 1.06, 1.08).	19
Huang (2022)	Global	PM_{10} $PM_{2.5}$ PM1 O_3 NO_2	683,081	Meta-analysis analyzing x studies examining children and adolescents	Air pollutants were correlated with childhood obesity and weight gain. For obesity, the association was considerable for PM_{10} (OR = 1.12, 95% CI: 1.06, 1.18), $PM_{2.5}$ (OR = 1.28, 95% CI: 1.13, 1.45), PM1 (OR = 1.41, 95% CI: 1.30, 1.53), and NO_2 (OR = 1.11, 95% CI: 1.06, 1.18). BMI status increased by OR = 0.08 (95% CI: 0.03, 0.12), OR = 0.11 (95% CI: 0.05, 0.17), and OR = 0.03 (95% CI: 0.01, 0.04) kg/m2 with 10 µg/m3	20

					increment in exposure to PM_{10}, $PM_{2.5}$, and NO_2.	
Chen (2023)	Taiwan	$PM_{2.5}$ NO_2	93,771	Cohort analysis of participants from the MJ Health Database, a longitudinal, population-based health research cohort	10-μg/m3 increases in $PM_{2.5}$ concentration were associated with increased adjusted risk of obesity (HR=1.07; 95% CI: 1.01, 1.14), hypertriglyceridemia (HR=1.17; 95% CI: 1.11, 1.23), low HDL-C (HR=1.09; 95% CI: 1.02, 1.17), hypertension (HR=1.15; 95% CI: 1.09, 1.21), and elevated fasting blood glucose (HR=1.15; 95% CI: 1.10, 1.20).	21
**Diabetes Related Mortality**						
Pope (2015)	USA	$PM_{2.5}$	669,046	Cohort analysis consisting of participants of the American Cancer Society Cancer Prevention Study II cohort.	$PM_{2.5}$exposure was associated with CVD mortality, HR = 1.12 (95% CI: 1.10, 1.15) per 10 μg/m3 increase in $PM_{2.5}$. $PM_{2.5}$ was also linked with diabetes and hypertension mortality.	22
Turner (2016)	USA	O_3 $PM_{2.5}$ NO_2	669,046	Large prospective cohort study consisting of the Cancer Prevention Study II participants enrolled in 1982.	O_3 was associated with all cause mortality (per 10 ppb, HR = 1.02 (95%CI: 1.01, 1.04)), cardiovascular disease mortality (per 10 ppb; HR = 1.03; (95%CI: 1.01, 1.06)), ischemic heart disease (HR = 1.06 (95% CI: 1.02, 1.09)), respiratory disease (HR = 1.04 (95%CI: 1.00, 1.09)), and chronic obstructive pulmonary disease (HR = 1.09 (95%CI: 1.03, 1.15)) in single-pollutant models.	23
Yin (2017)	China	$PM_{2.5}$	189,793	Prospective cohort study of men >40 years of age.	Found association between mortality per 10-μg/m3 increase in $PM_{2.5}$ concentration for nonaccidental causes (HR=1.09; 95% CI: 1.08, 1.09), CVD (HR=1.09; 95% CI: 1.08, 1.10), COPD (HR=1.12; 95% CI: 1.10, 1.13), and lung cancer (HR=1.12; 95% CI: 1.07, 1.14)	24
Bowe (2018)	USA, Global	$PM_{2.5}$	1,729,108	Longitudinal cohort study consisting of US veterans with no prior history of DM. Additionally, analyzed data from the Global Burden of Disease study.	A 10 μg/m3 increase in $PM_{2.5}$ was associated with increased risk of diabetes (HR=1.15, 95% CI: 1.08, 1.22). $PM_{2.5}$ was associated with increased mortality risk (HR=1.08, 95% CI: 1.03, 1.13).	25

					Globally, ambient $PM_{2.5}$ contributed to ~3.2 million (95% UI: 2.2, 3,8) incident cases of diabetes, ~8.2 million (95% UI: 5.8, 11.0) DALYs caused by diabetes, and 206,105 (95% UI 153,408, 259,119) deaths from diabetes attributable to $PM_{2.5}$ exposure.	

Abbreviations:CI: confidence interval; RR: risk-ratio; HR: hazard-ratio; EC: elemental carbon; UFP: ultra-fine particles; HOMA-IR: Homeostatic Model Assessment of Insulin Resistance; HRV: heart rate variability; SDNN: standard deviation of normal to normal R-R interval; HDL: High-Density Lipoprotein; LDL: low-density lipoprotein; BP: blood pressure; SBP: systolic blood pressure; DBP: diastolic blood pressure; DI: disposition index; 2hPG: 2 hour post-meal plasma glucose

REFERENCES

1. Brook RD, Xu X, Bard RL, et al. Reduced metabolic insulin sensitivity following sub-acute exposures to low levels of ambient fine particulate matter air pollution. *Sci Total Env*. 2013;448:66-71. doi:10.1016/j.scitotenv.2012.07.034

2. Brook RD, Sun Z, Brook JR, et al. Extreme Air Pollution Conditions Adversely Affect Blood Pressure and Insulin Resistance: The Air Pollution and Cardiometabolic Disease Study. *Hypertension*. 2016;67(1):77-85. doi:10.1161/HYPERTENSIONAHA.115.06237

3. Chen Z, Salam MT, Toledo-Corral C, et al. Ambient Air Pollutants Have Adverse Effects on Insulin and Glucose Homeostasis in Mexican Americans. *Diabetes Care*. 2016;39(4):547-554. doi:10.2337/dc15-1795

4. Thiering E, Cyrys J, Kratzsch J, et al. Long-term exposure to traffic-related air pollution and insulin resistance in children: results from the GINIplus and LISAplus birth cohorts. *Diabetologia*. 2013;56(8):1696-1704. doi:10.1007/s00125-013-2925-x

5. Wolf K, Popp A, Schneider A, et al. Association Between Long-term Exposure to Air Pollution and Biomarkers Related to Insulin Resistance, Subclinical Inflammation, and Adipokines. *Diabetes*. 2016;65(11):3314-3326. doi:10.2337/db15-1567

6. Khafaie MA, Salvi SS, Yajnik CS, Ojha A, Khafaie B, Gore SD. Air pollution and respiratory health among diabetic and non-diabetic subjects in Pune, India-results from the Wellcome Trust Genetic Study. *Environ Sci Pollut Res Int*. 2017;24(18):15538-15546. doi:10.1007/s11356-017-9148-5

7. Dang J, Yang M, Zhang X, et al. Associations of Exposure to Air Pollution with Insulin Resistance: A Systematic Review and Meta-Analysis. *Int J Environ Res Public Health*. 2018;15(11):2593. doi:10.3390/ijerph15112593

8. Zhang S, Mwiberi S, Pickford R, et al. Longitudinal associations between ambient air pollution and insulin sensitivity: results from the KORA cohort study. *Lancet Planet Health*. 2021;5(1):e39-e49. doi:10.1016/S2542-5196(20)30275-8

9. Zhan M, Li Z, Li X, Tao B, Zhang Q, Wang J. Effect of short-term ambient PM(2.5) exposure on fasting blood glucose levels: A longitudinal study among 47,471 people in eastern China. *Env Pollut*. 2021;290:117983. doi:10.1016/j.envpol.2021.117983

10. Eze IC, Hemkens LG, Bucher HC, et al. Association between ambient air pollution and diabetes mellitus in Europe and North America: systematic review and meta-analysis. *Env Health Perspect*. 2015;123(5):381-389. doi:10.1289/ehp.1307823

11. Kutlar Joss M, Boogaard H, Samoli E, et al. Long-Term Exposure to Traffic-Related Air Pollution and Diabetes: A Systematic Review and Meta-Analysis. *Int J Public Health*. 2023;68:1605718. doi:10.3389/ijph.2023.1605718

12. Sorensen M, Poulsen AH, Hvidtfeldt UA, et al. Effects of Sociodemographic Characteristics, Comorbidity, and Coexposures on the Association between Air Pollution and Type 2 Diabetes: A Nationwide Cohort Study. *Env Health Perspect*. 2023;131(2):27008. doi:10.1289/EHP11347

13. Liang W, Zhu H, Xu J, et al. Ambient air pollution and gestational diabetes mellitus: An updated systematic review and meta-analysis. *Ecotoxicol Env Saf*. 2023;255:114802. doi:10.1016/j.ecoenv.2023.114802

14. Matthiessen C, Lucht S, Hennig F, et al. Long-term exposure to airborne particulate matter and NO(2) and prevalent and incident metabolic syndrome - Results from the Heinz Nixdorf Recall Study. *Env Int*. 2018;116:74-82. doi:10.1016/j.envint.2018.02.035

15. Lee S, Park H, Kim S, et al. Fine particulate matter and incidence of metabolic syndrome in non-CVD patients: A nationwide population-based cohort study. *Int J Hyg Env Health*. 2019;222(3):533-540. doi:10.1016/j.ijheh.2019.01.010

16. Kim JS, Chen Z, Alderete TL, et al. Associations of air pollution, obesity and cardiometabolic health in young adults: The Meta-AIR study. *Env Int*. 2019;133(Pt A):105180. doi:10.1016/j.envint.2019.105180

17. Li X, Wang M, Song Y, et al. Obesity and the relation between joint exposure to ambient air pollutants and incident type 2 diabetes: A cohort study in UK Biobank. *PLoS Med*. 2021;18(8):e1003767. doi:10.1371/journal.pmed.1003767

18. Voss S, Schneider A, Huth C, et al. ENVINT-D-20-01309: Long-term exposure to air pollution, road traffic noise, residential greenness, and prevalent and incident metabolic syndrome: Results from the population-based KORA F4/FF4 cohort in Augsburg, Germany. *Env Int*. 2021;147:106364. doi:10.1016/j.envint.2020.106364

19. Bowe B, Gibson AK, Xie Y, et al. Ambient Fine Particulate Matter Air Pollution and Risk of Weight Gain and Obesity in United States Veterans: An Observational Cohort Study. *Environ Health Perspect*. 2021;129(4):047003. doi:10.1289/EHP7944

20. Huang C, Li C, Zhao F, Zhu J, Wang S, Sun G. The Association between Childhood Exposure to Ambient Air Pollution and Obesity: A Systematic Review and Meta-Analysis. *Int J Env Res Public Health*. 2022;19(8). doi:10.3390/ijerph19084491

21. Chen YC, Chin WS, Pan SC, Wu CD, Guo YL. Long-Term Exposure to Air Pollution and the Occurrence of Metabolic Syndrome and Its Components in Taiwan. *Env Health Perspect*. 2023;131(1):17001. doi:10.1289/EHP10611

22. Pope CA, Turner MC, Burnett RT, et al. Relationships between fine particulate air pollution, cardiometabolic disorders, and cardiovascular mortality. *Circ Res*. 2015;116(1):108-115. doi:10.1161/CIRCRESAHA.116.305060

23. Turner MC, Jerrett M, Pope CA, et al. Long-Term Ozone Exposure and Mortality in a Large Prospective Study. *Am J Respir Crit Care Med*. 2016;193(10):1134-1142. doi:10.1164/rccm.201508-1633OC

24. Yin P, Brauer M, Cohen A, et al. Long-term Fine Particulate Matter Exposure and Nonaccidental and Cause-specific Mortality in a Large National Cohort of Chinese Men. *Environ Health Perspect*. 2017;125(11):117002. doi:10.1289/EHP1673

25. Bowe B, Xie Y, Li T, Yan Y, Xian H, Al-Aly Z. The 2016 global and national burden of diabetes mellitus attributable to PM2·5 air pollution. *Lancet Planet Health*. 2018;2(7):e301-e312. doi:10.1016/S2542-5196(18)30140-2
